# Increased risk of knee osteoarthritis in patients using oral N-acetylcysteine: a nationwide cohort study

**DOI:** 10.1186/s12891-020-03562-1

**Published:** 2020-08-10

**Authors:** Ying-Ting Yeh, Chung-Chao Liang, Chia-Ling Chang, Chung-Y. Hsu, Pei-Chen Li

**Affiliations:** 1 Department of Physical Medicine and Rehabilitation Medicine, Hualien Tzu Chi Hospital, Buddhist Tzu Chi Medical Foundation, Hualien, Taiwan; 2 Management Office for Health Data, China Medical University Hospital, Taichung, Taiwan; 3 Graduate Institute of Clinical Medical Science, China Medical University, Taichung, Taiwan; 4 Department of Obstetrics and Gynecology, Hualien Tzu Chi Hospital, Buddhist Tzu Chi Medical Foundation, Hualien, Taiwan

**Keywords:** Knee osteoarthritis, N-acetylcysteine, Knee, Cartilage

## Abstract

**Background:**

Knee osteoarthritis (OA) is known to be a progressive degenerative disorder; however, recent evidence suggests that inflammatory mediators contribute to cartilage degradation. Studies have reported that N-acetylcysteine (NAC) had a promising effect on the reduction of the synthesis of proinflammatory and structural mediators by synovial cells. Given the lack of relevant clinical trials, we conducted this study to determine the relationship between NAC use and risk of knee OA.

**Methods:**

We designed a retrospective cohort study from 2000 to 2013. Patients who received oral NAC over 28 days within 1 year after the first prescription were defined as the case group, whereas those without NAC use were considered as candidates of the control group. We adopted 1:4 propensity-score matching by age, sex, index year, and comorbidities to obtain the control group. The primary outcome was a new diagnosis of knee OA during the follow-up period.

**Results:**

Our study sample comprised 12,928 people who used NAC and 51,715 NAC nonusers. NAC users had a significantly higher incidence of osteoarthritis (adjusted hazard ratio: 1.42, *P* < .001) than did NAC nonusers. Also, in analyses stratified by age group and sex, all subgroups exhibited a significantly higher incidence of knee osteoarthritis (*P* < .0001) among NAC users than among NAC nonusers. The use of oral NAC was associated with nearly four-fold increased the risk of knee OA in the young age group.

**Conclusions:**

Long-term use of oral NAC is associated with a higher risk of knee OA.

## Background

Osteoarthritis (OA) is one of the most common degenerative diseases that affects the elderly population, leading to disability and reduced quality of life [1, 2]. OA affects at least 27 million adults in the United States annually, accounting for health care costs of more than US$185 billion. Knee and hip OA are the two most prevalent types of OA in the elderly population [3]. OA is characterized by the disruption of articular cartilage and loss of viscoelastic properties of the synovial fluid [4]. OA has been classified as noninflammatory arthritis because of the presence of fewer leukocytes in the synovial fluid than that observed in rheumatoid arthritis, reactive arthritis, and septic arthritis [5]. However, an increasing body of evidence suggests that synovial inflammation and proinflammatory cytokines are crucial to the pathogenesis of OA. Posttraumatic or spontaneous inflammatory chemokine-mediated pathways accelerate the progression of OA, resulting in joint-tissue destruction and remodeling [6]. Hence, new biological products that focus on reducing synovial inflammation were introduced for knee OA treatment, such as hyaluronic acid (HA) and platelet-rich plasma.

N-acetylcysteine (NAC), a cysteine prodrug and glutathione precursor, has been used in therapeutic practices for several decades [7], such as in mucolytic medication in chronic lung disease, as an antidote for acetaminophen intoxication, as a renal protective agent for contrast-induced nephropathy [8], and as an antioxidant for hypoxic-ischemic brain injury in stroke [9]. NAC was found to have protective effects against oxidative stress in many organs. According to experimental and tissue culture studies, NAC is effective in clearing free-oxygen radicals, slowing the cartilage degradation process, reducing synovial inflammation, and reducing pain. In addition, one recent study confirmed that NAC use in patients with knee OA yielded more effective pain relief and better functional outcomes compared with intra-articular injection of HA [10]. However, few studies have examined the therapeutic effects of NAC on OA.

Knee OA is a highly prevalent disease worldwide, which results in ambulation disability and multiple comorbidities. Previous studies suggested NAC, a frequently used medication exhibits potential antioxidant benefits. The aim of the present study is to investigate the risk of knee OA in patients using NAC.

## Methods

### Data sources

Taiwan instituted the National Health Insurance (NHI) system in 1995 to provide overall health care coverage to its people, with a coverage rate of 99% among clinics and hospitals by 1996 [11]. Most of Taiwan’s population receives health care through the NHI. All data related to these services were collected and input into the National Health Insurance Research Database (NHIRD), which was operated by the National Health Research Institutes from 1997 to 2013. The NHIRD includes ambulatory care, inpatient care, and registration data of the individuals covered by the NHI. In this study, we used the longitudinal health insurance database 2000 (LHID 2000). The LHID 2000 contains data of 1,000,000 participants randomly selected from among the 23 million enrollees of the NHI program in 2000. No statistical significance in terms of age, sex, and medical costs were observed among the sample groups. All the databases can be interlinked through individual PIN codes. Ambulatory care claims contain the individual dates of visits and International Classification of Diseases, Ninth Revision, Clinical Modification (ICD-9-CM) codes. Outpatient claims contain the ICD-9-CM codes, including one primary diagnosis and two secondary diagnoses; Inpatient claims contain the ICD-9-CM codes from primary diagnosis and up to 4 secondary diagnoses. Because the NHIRD contains de-identified secondary data for research, informed consent was waived for the present study. This study was approved by the Institutional Review Board of China Medical University Hospital [CMUH104-REC2–115(CR-2)].

### Study population

**Fig. 1:**
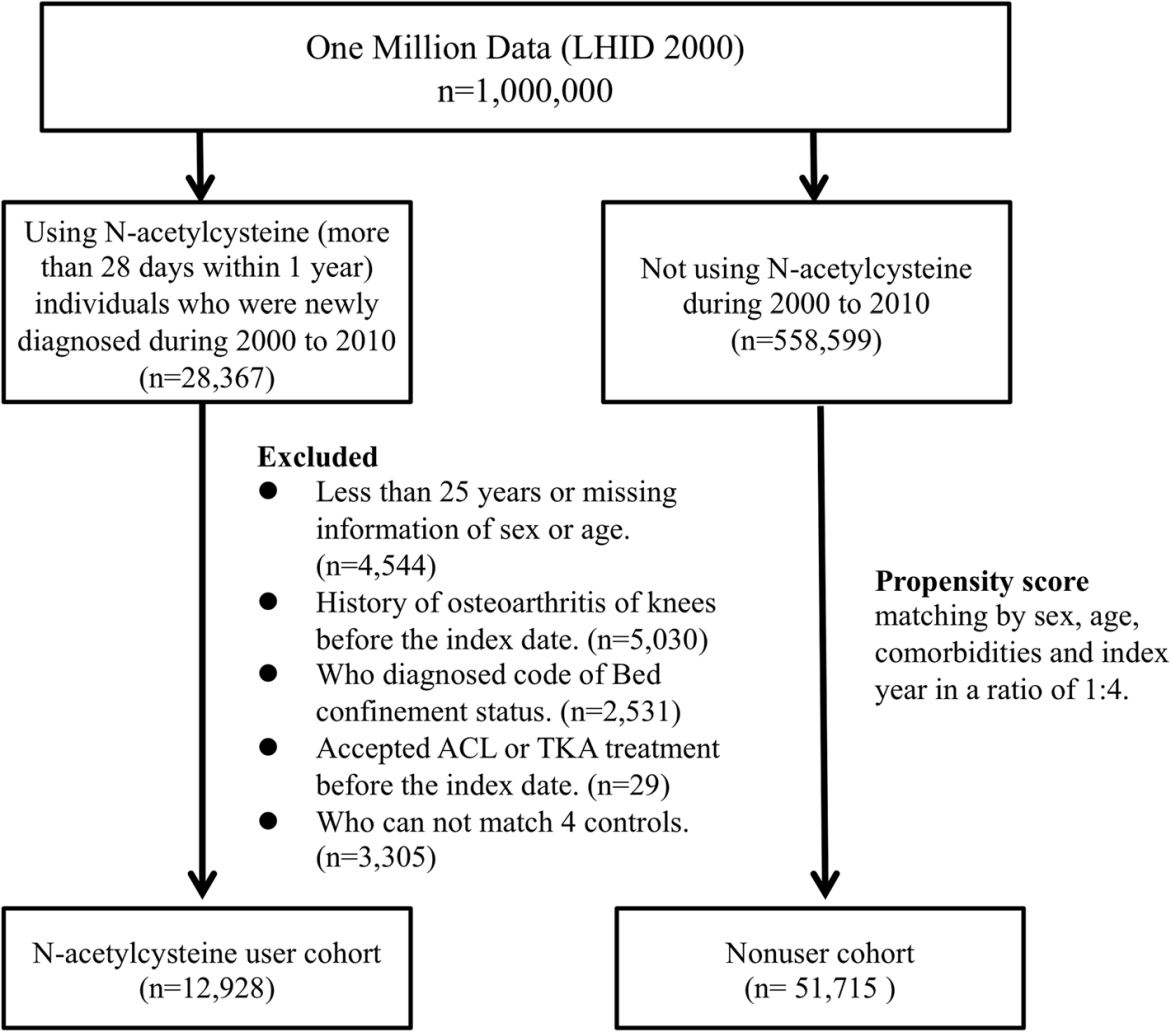
Flow chart for establishing N-acetylcysteine user cohort and controls

This retrospective cohort study included patients aged older than 25 years who received oral NAC between 2000 and 2013. Patients who received oral NAC for more than 28 days within 1 year were considered as NAC users. The patients in the comparison group were nonusers selected from the database. The first day of NAC use was considered the index date. Patients with a history of knee operation (e.g., total knee arthroplasty and anterior cruciate ligament reconstruction) before the index date, a history of knee OA before the index date, and a bed confinement status (ICD-9-CM: V48.84; or had been living in nursing care) were excluded. We did not exclude those with a diagnosis of rheumatoid arthritis, septic arthritis, or aseptic arthritis but considered them as covariates. It is possible to be diagnosed with knee OA and other inflammatory arthritis concurrently. The selection algorithm is shown in Fig. 1.

### Outcome variable

The primary outcome was the occurrence of knee OA, including generalized (ICD-9-CM: 715.00, 715.80, 715.89), localized (ICD-9-CM: 715.16, 715.36), and unspecified (ICD-9-CM: 715.96), defined as at least two initial outpatient visits or at least one hospital admission for knee OA. Patients in both cohorts were followed up until December 31, 2013, withdrawal from the NHI program; or the occurrence of knee OA.

### Demographic and comorbidity profiles

Comorbidities of knee OA comprised myocardial infarction (ICD-9-CM: 410 and 412), congestive heart failure (ICD-9-CM: 428), cerebral vascular disease (ICD-9-CM: 430–438), rheumatoid arthritis (ICD-9-CM: 714.0), systemic lupus erythematosus (ICD-9-CM: 710.0), diabetes mellitus (ICD-9-CM: 250), hypertension (ICD-9-CM: 401–405), gout (ICD-9-CM: 274), septic arthritis (ICD-9-CM: 711), aseptic necrosis (ICD-9-CM: 733.4), chronic obstructive pulmonary disease (COPD; ICD-9-CM: 490, 491, 492, 494, and 496), obesity (ICD-9-CM: 278), osteoarthritis of hand (ICD-9-CM: 715.14) and previous knee, leg, ankle, and foot injury (ICD-9-CM: 959.7). Sociodemographic factors included age and sex. Age was divided into three groups: 25–39 years, 40–64 years, and older than 65 years.

### Statistical analysis

We compared the baseline characteristics of the NAC sample and comparison cohorts by using standardized mean differences (SMD), with an SMD of < 0.1 indicating a negligible difference in mean values or proportions between the two cohorts. We used propensity-score matching by age, sex, index year, and all comorbidities to obtain the two cohort groups [12, 13]. The Cox proportional hazards model was used to estimate the hazard ratios (HRs) and 95% confidence intervals (95% CIs) of knee OA, and sex, age, and comorbidities were adjusted in the model. The difference between the two cohorts in the development of knee OA was estimated using the Kaplan–Meier method and log-rank test.

Statistical analysis was performed using SAS 9.4 (SAS Institute, Cary, NC, USA) and R software (R Foundation for Statistical Computing, Vienna, Austria), respectively. *P* < .05 in two-tailed tests indicated statistical significance.

## Results

By using the NHIRD, we identified 28,367 NAC users and 558,599 nonusers. After propensity-score matching, we obtained 12,928 and 51,715 patients in the cohort and comparison groups, respectively.

The baseline characteristics were shown in Table 1, and the distribution of sex, age, and comorbidities had a similar effect size in the two groups. The proportion of NAC use was higher in men than in women. Patients aged 65 years or older had the highest percentage of NAC use. The mean (SD) age was 56.3 (15.00) and 56.9 (13.7) years for the cohort and comparison groups, respectively. The mean (median) follow-up period was 5.30 (4.80) and 6.50 (6.01) years for the NAC and comparison groups, respectively.

**Table 1:** Demographic characteristics of the patients used and non-used N-acetylcysteine in Taiwan

Variable	N-acetylcysteine used^a^		Standardized mean difference^b^
	No	Yes	
	***n*** = 51,715	***n*** = 12,928	
	N (%)	N (%)	
**Sex**			
Female	23,379 (45.2)	5838 (45.2)	0.001
Male	28,336 (54.8)	7090 (54.8)	0.001
**Age at baseline, years**			
25–39 years	1718 (3.3)	485 (3.8)	0.023
40–64 years	34,088 (65.9)	8578 (66.4)	0.009
Older than 65 years	15,909 (30.8)	3865 (29.9)	0.019
Mean (SMD)			
**Comorbidities**			
Myocardial infarction	776 (1.5)	239 (1.9)	0.027
Congestive heart failure	2109 (4.1)	665 (5.1)	0.051
Cerebral vascular disease	6447 (12.5)	1739 (13.5)	0.029
Rheumatoid arthritis	790 (1.5)	201 (1.6)	0.002
Systemic lupus erythematosus	267 (0.5)	73 (0.6)	0.007
Diabetes mellitus	10,997 (21.3)	2805 (21.7)	0.011
Hypertension	23,911 (46.2)	5850 (45.3)	0.020
Gout	7109 (13.8)	1832 (14.2)	0.012
Septic arthritis	156 (0.3)	50 (0.4)	0.015
Aseptic necrosis	234 (0.5)	57 (0.4)	0.002
COPD	13,814 (26.7)	3647 (28.2)	0.034
Obesity	463 (0.9)	116 (0.9)	< 0.001
Osteoarthritis of hand	36 (0.1)	9 (0.1)	< 0.001
Previous knee, leg, ankle, and foot injury	113 (0.2)	21 (0.2)	0.013

^a^ The means (median) of follow-up period were 5.3 (4.8) years and 6.5 (6.01) years for N-acetylcysteine cohort group and compared cohort group

^b^ An SMD (standardized mean difference) of ≤0.1 indicates a negligible difference between the two cohorts

Total 8240 patients developed knee OA during follow-up. The overall incidence density of knee OA was higher in the NAC cohort than in the controls (6406 events per 69,351.19 person-years vs. 1834 events per 337,321.5 person-years). Univariate and multivariate analyses by using the Cox proportional hazards model revealed that the crude HR of knee OA was 1.39 (95% CI: 1.32–1.46, *p* < .001) in the NAC cohort compared with the nonuser cohort. After adjustment for sex, age, and comorbidities, the adjusted HR (aHR) was 1.42 (95% CI: 1.35–1.49, *p* < .001). Men had a lower incidence rate of knee OA than did women (aHR: 0.56, 95% CI: 0.53–0.58, *p* < .001). Patients aged 25–39 years and 40–64 years had a lower incidence rate of knee OA than did those older than 65 years. Regarding comorbidities, cerebral vascular disease (aHR: 1.11, 95% CI: 1.04–1.18, *p* = 0.001), rheumatoid arthritis (aHR: 1.57, 95% CI: 1.37–1.80, *p* < 0.001), diabetes mellitus (aHR: 1.12, 95% CI: 1.06–1.17, *p* < 0.001), hypertension (aHR: 1.52, 95% CI: 1.45–1.60, *p* < 0.001), gout (aHR: 1.39, 95% CI: 1.31–1.47, *p* < 0.001), and COPD (aHR: 1.31, 95% CI: 1.25–1.37, *p* < 0.001) might be potential risk factors for the incidence of knee OA (Table 2).

**Table 2:** Cox model with hazard ratios and 95% confidence intervals of osteoarthritis of knees associated with N-acetylcysteine used

Variable	Osteoarthritis of knees	Crude^**a**^		Adjusted^**b**^	
	no. (***n*** = 8240)	HR (95%CI)	***p***-value	HR (95%CI)	***p***-value
**N-acetylcysteine used**					
No	6406	reference		reference	
Yes	1834	1.39 (1.32–1.46)	< 0.001	1.42 (1.35–1.49)	< 0.001
**Sex**					
Female	4858	reference		reference	
Male	3382	0.58 (0.55–0.61)	< 0.001	0.56 (0.53–0.58)	< 0.001
**Age at baseline, years**					
25–39 years	36	0.06 (0.05–0.09)	< 0.001	0.09 (0.07–0.13)	< 0.001
40–64 years	4663	0.47 (0.45–0.49)	< 0.001	0.54 (0.52–0.57)	< 0.001
Older than 65 years	8113	reference		reference	
**Comorbidities**					
Myocardial infarction	127	1.19 (1.00–1.42)	0.049	0.94 (0.79–1.13)	0.52
Congestive heart failure	429	1.62 (1.47–1.78)	< 0.001	1.06 (0.96–1.17)	0.261
Cerebral vascular disease	1299	1.61 (1.52–1.71)	< 0.001	1.11 (1.04–1.18)	0.001
Rheumatoid arthritis	216	1.95 (1.70–2.23)	< 0.001	1.57 (1.37–1.80)	< 0.001
Systemic lupus erythematosus	35	0.81 (0.58–1.14)	0.226	0.78 (0.56–1.09)	0.151
Diabetes mellitus	2142	1.49 (1.42–1.57)	< 0.001	1.12 (1.06–1.17)	< 0.001
Hypertension	4909	2.04 (1.95–2.13)	< 0.001	1.52 (1.45–1.60)	< 0.001
Gout	1489	1.53 (1.44–1.62)	< 0.001	1.39 (1.31–1.47)	< 0.001
Septic arthritis	35	1.63 (1.17–2.27)	0.004	1.31 (0.94–1.83)	0.108
Aseptic necrosis	38	1.19 (0.87–1.64)	0.278	1.37 (0.99–1.88)	0.055
COPD	2587	1.32 (1.26–1.38)	< 0.001	1.31 (1.25–1.37)	< 0.001
Obesity	79	1.16 (0.93–1.45)	0.186	1.03 (0.82–1.29)	0.796
Osteoarthritis of hand	4	0.80 (0.30–2.14)	0.663	0.72 (0.27–1.92)	0.514
Previous knee, leg, ankle, and foot injury	20	1.37 (0.88–2.13)	0.157	1.42 (0.91–2.20)	0.118

*Crude HR*^a^ represented relative hazard ratio, *Adjusted HR*^b^ represented adjusted hazard ratio: mutually adjusted for N-acetylcysteine used, age, gender and baseline comorbidities (as like tables) in Cox proportional hazard regression

**Fig. 2:**
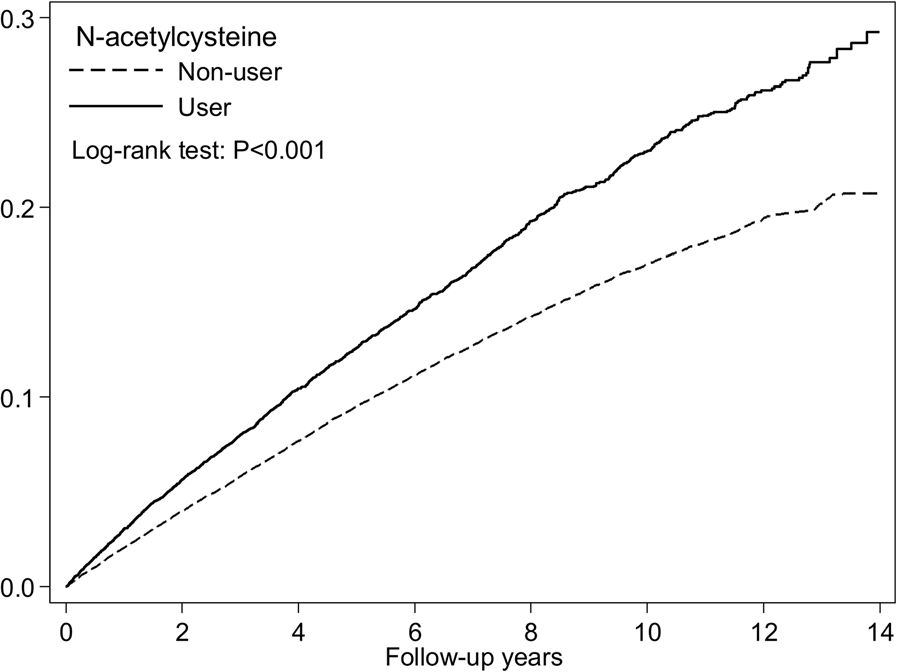
Cumulative incidence of knee osteoarthritis between the N-acetylcysteine user and nonuser cohorts through Kaplan–Meier analysis. (Log-rank test, *P* < .0001)

Table 3 presents the subgroup analysis by sex and age groups. Stratified by sex, male patients had a lower incidence rate of knee OA than did female patients in both cohorts. The adjusted HRs were 1.39 and 1.43 for women and men, respectively. The incidence rate of knee OA was higher in the NAC cohort than in the comparison group among patients in the age groups 25–39 years, 40–64 years, and older than 65 years. The young age group had a 4-fold increased risk of knee OA after taking NAC (aHR: 3.98, 95% CI: 2.03–7.79, *p* < .001). The Kaplan–Meier analysis revealed that the cumulative incidence curves of knee OA were significantly higher in the NAC cohort than in the control cohort (log-rank test, *P* < .0001) (Fig. 2).

**Table 3:** Incidence and Cox proportional hazard regression with hazard ratios and 95% confidence intervals of osteoarthritis of knees associated with used N-acetylcysteine drug by gender and age group

Variable	N-acetylcysteine used						N-acetylcysteine used vs. non-N-acetylcysteine used			
	No			Yes			Crude		Adjusted HR^b^	
	Event	Person years	IR^**a**^	Event	Person years	IR^**a**^	HR (95%CI)	***p***-value	HR (95%CI)	***p***-value
**Total**	6406	337,321.5	1.90	1834	69,351.19	2.64	1.39 (1.32–1.46)	< 0.001	1.42 (1.35–1.49)	< 0.001
**Gender**										
Female	3766	151,082.4	2.49	1092	33,775.32	3.23	1.30 (1.21–1.39)	< 0.001	1.39 (1.30–1.49)	< 0.001
Male	2640	186,239	1.42	742	35,575.87	2.09	1.47 (1.35–1.59)	< 0.001	1.43 (1.32–1.55)	< 0.001
**Age group, year**										
25–39 years	18	13,345.57	0.13	18	3375.513	0.53	3.89 (2.02–7.48)	< 0.001	3.98 (2.03–7.79)	< 0.001
40–64 years	3516	237,534.4	1.48	1147	50,744.04	2.26	1.53 (1.43–1.64)	< 0.001	1.44 (1.35–1.54)	< 0.001
Older than 65 years	2872	86,441.49	3.32	669	15,231.64	4.39	1.31 (1.20–1.43)	< 0.001	1.33 (1.22–1.44)	< 0.001

*Abbreviations*: ^a^ *IR* Incidence rates, per 100 person-years, *HR* Hazard ratio, *CI* Confidence interval

^b^ represented adjusted hazard ratio: mutually adjusted for N-acetylcysteine used, age, gender and baseline comorbidities (as like Table 2) in Cox proportional hazard regression

## Discussion

A growing body of evidence suggests that both local and systemic inflammatory processes mediated by the innate immune system contribute to the evolution of knee OA [14, 15]. Several antioxidant agents are used to control the inflammatory response. NAC inhibits the inflammatory cytokines TNFα, IL-1β, and IL-6 in lipopolysaccharide (LPS)-activated macrophages and also modulates COX-2 expression [16, 17]. Recent in vivo and ex vivo studies by Riegger J et al. also suggested NAC exhibits antioxidative effects and alleviates cartilage degeneration after trauma [18, 19]. Hence, we hypothesized that NAC presents anti-inflammatory effects, which would reduce damage to the articular cartilage and synovial joint. However, our findings showed that patients taking NAC for more than 28 days per year had a significantly higher risk of knee OA. Furthermore, the effects remained significant in all subgroups of different sexes or ages after stratification analysis.

One possible explanation for these contradictory results is that NAC’s anti-inflammatory properties have been proven previously only by in vitro studies [17, 18, 20, 21]. The effect of NAC on circulating cytokines or the local impact on the joints and cartilage has not previously been examined. The exact pathophysiology underlying the increased risk of knee OA caused by long-term use of NAC remains unknown. Because chondrocyte activation plays a crucial role in OA, multiple intracellular signals modulate cell activity by regulating cell survival and programming cell death [22]. The critical factor p53 triggers DNA repair and causes apoptosis of chondrocytes [23]. Moreover, p53 expression was found to be significantly decreased in OA cartilages [22]. However, antioxidants, including NAC, reduced p53 expression in an in vivo study on rats and even increased tumor cell proliferation [24]. Regarding the hypothesis that NAC retards the inflammation process, long-term treatment with oral NAC was found to cause no significant reduction in the activity of neutrophilic inflammatory biomarkers in patients with cystic fibrosis [25]. However, in a pilot study, Ozcamdalli compared the efficacies between intra-articular injections of HA or NAC in the treatment of knee OA and revealed that NAC had outcomes comparable to those of HA in terms of pain and function scales, but biomarkers within or between groups exhibited equivocal results [10].

Age, obesity, previous knee injury, female sex, muscle weakness, joint laxity, and hand OA are known risk factors for knee OA [1, 2]. Our results showed that female sex, old age, cerebral vascular disease, rheumatoid arthritis, diabetes mellitus, hypertension, gout, and COPD are positively correlated with knee OA. A pilot study by Howard et al. suggested that gout is associated with increased prevalence and severity of knee OA (adjusted odds ratio [OR]: 3.80) [26]. Inflammatory arthritis can occur concurrently or subsequently with knee OA. In addition, patients with metabolic syndrome are more susceptible to knee OA. A recent meta-analysis found that metabolic syndrome increased the risk of knee OA (crude pooled OR 2.24, 95% CI: 1.38–3.64) in 8 studies [27]. Increasing evidence suggests a shared mechanism between OA and metabolic syndrome, including inflammation, oxidative stress, and endothelial dysfunction [28, 29]. Another study found that OA not only causes pain or disability but also increases the risk of death due to cardiovascular disease [21]. Additionally, NAC has been widely used in clinical practice, particularly in patients with chronic diseases such as chronic bronchitis, cystic fibrosis, and other pulmonary disorders [25]. This population is highly susceptible to obstructed airways due to sticky sputum, which can cause decreased lung function and a higher risk of respiratory infections. A systematic review by Wshah et al. implied that the prevalence of OA is high among individuals with COPD [30]. Both COPD and OA are associated with chronic systemic inflammation and limited physical activity, and the co-occurrence of these conditions is a crucial concern. Our finding has important implications for the early prevention of and intervention for knee OA in this population. Patients who require long-term NAC use should be informed and advised about the risk of knee OA.

Our study was a large number, and 14 years of a population-based cohort study to investigate the relationship between long-term NAC use and the risk of knee OA, and the large sample size yielded reliable and accurate results. Prevalence-incidence bias or selection bias could be minimized in the cohort study. Our study adjusted for independent variables, such as obesity, gout, and hypertension, known to influence the risk of knee OA. We also excluded bedridden patients and populations under nursing home care. Diagnosis of knee OA in bedridden patients may be inaccurate due to their reduced expression of knee pain or because of the low weight being borne on their knee joint. Additionally, joint constriction might generate uncertain factors for knee OA diagnosis. However, the study had several potential limitations. First, the severity and symptoms of knee OA were not assessed because of the unavailability of NHIRD data. Moreover, because data on the dose and administration forms of oral NAC (e.g., oral granule or oral effervescent tablet) were not available in the NHIRD, the dose-response relationship could not be determined. Besides, the cases of knee OA were identified by ICD-9-CM codes instead of validated by medical records. However, all insurance claims should be scrutinized by medical reimbursement specialists and peer review according to the standard diagnosed criteria in the study. Therefore, the diagnoses based on ICD-9 codes in this study should be highly reliable. The relatively homogenous population living in Taiwan increases the internal validity and reliability of the findings, but the ability to generalize our findings to other populations is unknown.

## Conclusions

In our study, the findings indicate that long-term use of oral NAC is positively related to a higher risk of knee OA. We were unable to identify the apparent benefits of NAC use for protection against knee OA. Further prospective studies are warranted to confirm the study findings.

## Data Availability

The datasets generated and/or analysed during the current study are available from the corresponding author on reasonable request.

## Abbreviations

OA: Osteoarthritis
NAC: N-acetylcysteine;
HA: Hyaluronic acid
NHI: National Health Insurance
NHIRD: National Health Insurance Research Database
LHID: Longitudinal health insurance database
ICD-9-CM: International Classification of Diseases, Ninth Revision, Clinical Modification
COPD: Chronic obstructive pulmonary disease
SD: Standardized mean differences
HRs: Hazard ratios
CI: Confidence intervals
LPS: Lipopolysaccharide
